# Clinical and tissue evidence of immune dysregulation in osteosarcoma: altered peripheral neutrophil-related indices and reduced APOE expression

**DOI:** 10.3389/fimmu.2026.1753859

**Published:** 2026-05-07

**Authors:** Jichong Zhu, Chengqian Huang, Weiming Tan

**Affiliations:** 1 The First Affiliated Hospital of Guangxi Medical University, Nanning, China; 2 The Affiliated Hospital of Youjiang Medical University for Nationalities, Baise, China; 3 Key Laboratory of Molecular Pathology in Tumors of Guangxi Higher Education Institutions, Baise, China

**Keywords:** APOE, immune dysregulation, neutrophils, osteosarcoma, tumor microenvironment

## Abstract

**Background:**

Osteosarcoma (OS) is a common primary malignant bone tumor in children and adolescents. Increasing evidence suggests that immune dysregulation contributes to OS progression, but its tissue-level and systemic features remain incompletely defined. This study aimed to characterize immune-related alterations in OS and identify key immunoregulatory molecules associated with disease progression.

**Methods:**

Public transcriptomic datasets from the Gene Expression Omnibus (GSE14359, GSE19276, GSE21257, GSE28424, and GSE36001) were analyzed using differential expression, functional enrichment, protein–protein interaction analysis, immune-infiltration analysis, and machine-learning algorithms. Public single-cell RNA-seq data from six primary OS samples were additionally analyzed to define the cellular composition of the OS microenvironment and localize APOE expression. Peripheral blood parameters were retrospectively compared between 604 OS patients and 600 hospital-based non-malignant controls with cervical spondylosis. APOE expression was further evaluated by immunohistochemistry in paired tumor and adjacent non-tumor tissues.

**Results:**

Five core genes were identified, including APOE, CD74, RPL26L1, WDR12, and CXCL12, among which APOE was significantly downregulated in OS tissues. Immune-infiltration analysis suggested reduced neutrophils and CD8^+^ T cells with enrichment of M2 macrophages, consistent with an immunosuppressive microenvironment. Single-cell RNA-seq analysis of 22,997 cells identified 11 major cell populations, with macrophages and fibroblasts as prominent components. APOE expression was predominantly enriched in macrophages, with lower expression in fibroblasts and mesenchymal stem cells. In the peripheral blood analysis, OS patients showed altered neutrophil-related indices, characterized by lower absolute neutrophil counts but higher neutrophil percentages than controls. Immunohistochemistry further supported reduced APOE expression in OS tissues.

**Conclusions:**

This study integrates tissue-level, single-cell, and exploratory peripheral hematologic evidence to support immune-related alterations in OS. APOE downregulation and altered peripheral neutrophil-related indices may represent clinically relevant features associated with immune dysregulation in osteosarcoma.

## 1 Introduction

Osteosarcoma (OS) is one of the most common primary malignant bone tumors, predominantly affecting children and adolescents, and typically arising in the metaphyseal regions of long bones (1). Although advances in multimodal therapy have increased the 5-year survival rate to approximately 60–70%, the prognosis for patients with recurrence or metastasis remains poor, indicating limitations of current treatment strategies (2). Epidemiological studies show that, due to China’s large population, the number of new OS cases ranks among the highest worldwide (3). Recent studies have revealed that inflammatory responses and the immune microenvironment play crucial roles in the initiation and progression of OS, where alterations in immune cell composition and function may influence tumor growth and metastasis, highlighting the importance of immune characteristics in understanding its pathogenesis (4).

Despite continuous advances in OS management, its pronounced heterogeneity and immune-evasive characteristics remain major clinical challenges. Increasing evidence indicates that inflammatory responses and the tumor immune microenvironment play essential roles in OS progression, with alterations in tumor-associated immune cells—such as macrophages, T lymphocytes, and neutrophils—contributing to tumor growth and metastasis (5). However, most existing studies are based on limited tumor tissue samples and are often affected by cohort heterogeneity, leaving the systemic immune status of OS patients insufficiently characterized (6). As a readily accessible indicator of systemic immunity, peripheral blood parameters—particularly neutrophil levels—may reflect underlying immune dysregulation, yet comprehensive investigations in OS remain scarce (7). In parallel, studies on immune-regulatory molecules in OS have gained increasing attention (8). Molecules such as APOE have been implicated in inflammatory modulation and immune response, highlighting their potential value as biomarkers (9). Nonetheless, research integrating large-scale hematologic data with tissue-level validation is still limited, restricting the clinical translational potential of these findings. Therefore, elucidating systemic immune alterations and identifying key immune-modulatory molecules in OS are critical for advancing our understanding of its immunological mechanisms.

Given the current limitations in understanding the systemic immune characteristics and key immunoregulatory molecules in OS, we hypothesized that osteosarcoma exhibits a systemic immunosuppressive profile characterized by altered neutrophil-related hematologic indices and dysregulated expression of immune-related genes, particularly APOE. Based on this hypothesis, this study aimed to comprehensively evaluate immune alterations in OS by integrating transcriptomic analysis, machine-learning-based hub gene screening, immune-infiltration profiling, peripheral blood assessment, and tissue-level validation. Specifically, we first analyzed multiple public datasets to identify genes that were significantly dysregulated in OS tissues and to explore their potential biological functions and immune relevance. Given the growing evidence that immune-cell dysfunction and microenvironmental remodeling are central to osteosarcoma progression, we prespecified immune-related biological signals as a major interpretive focus for the downstream analyses. We then examined the association between candidate genes and immune-cell infiltration, with particular attention to neutrophil-related changes. To determine whether these alterations were also reflected systemically, we collected and analyzed complete blood count (CBC) data from more than 600 OS patients. Finally, the expression pattern of the key screened gene was validated in clinical tissue samples. In addition, to provide cell-type-level support for the tumor microenvironment findings derived from bulk transcriptomic data, we further analyzed public single-cell RNA-seq data from primary osteosarcoma samples to characterize major cellular components and to determine the cellular distribution of APOE. Through this multi-level design, we sought to provide convergent evidence for immune dysregulation in OS and to identify clinically relevant immunoregulatory molecules associated with this process.

## 2 Materials and methods

### 2.1 Core gene identification and integrated transcriptomic analysis

OS-related gene-expression datasets, including GSE14359, GSE19276, GSE21257, GSE28424, and GSE36001, were downloaded from the GEO database. For differential-expression analysis, GSE28424 and GSE36001 were merged after probe annotation and expression-matrix preprocessing. When multiple probes mapped to the same gene symbol, the average expression value was used. Expression data were log2-transformed when necessary and normalized prior to integration. Batch effects between datasets were adjusted using the ComBat algorithm in the sva package in R. Because the two datasets showed broadly comparable global expression distributions after preprocessing, this step was applied primarily to reduce potential residual batch effects before integrated downstream analysis. Boxplots and PCA before and after batch correction were used as visual assessments of cross-dataset comparability, and the revised PCA plots simultaneously annotated biological group and dataset source to improve interpretability.

Differentially expressed genes (DEGs) between OS and control samples were identified using the limma package with thresholds of |log2 fold change| ≥ 0.585 and P < 0.05. GO annotation and KEGG pathway enrichment analyses were then performed to explore the biological functions of the DEGs. Weighted gene co-expression network analysis (WGCNA) was further conducted to identify phenotype-related co-expression modules, and the intersecting genes between WGCNA modules and DEGs were considered candidate genes for downstream analyses.

For machine-learning analysis, GSE14359, GSE19276, and GSE21257 were integrated as the training cohort to identify hub genes capable of discriminating OS from non-tumor/control samples. Four algorithms, including Random Forest (RF), Support Vector Machine (SVM), eXtreme Gradient Boosting (XGB), and Generalized Linear Model (GLM), were applied and compared. Model performance was evaluated primarily based on discrimination ability using receiver operating characteristic (ROC) analysis and the corresponding area under the curve (AUC). The top-ranked genes from the best-performing models were intersected to identify robust hub genes. The machine-learning framework in this study was designed primarily for prioritization of robust hub genes across integrated public datasets, rather than for development of a final clinical prediction tool.

Batch effects between datasets were corrected using the ComBat function in the sva package in R. GSE28424 and GSE36001 were used as independent validation cohorts to assess the reproducibility of the selected genes and the performance of the model. Calibration curves were generated to evaluate the agreement between predicted and observed classification probabilities, and decision curve analysis (DCA) was used to estimate the potential net clinical benefit of the nomogram across a range of threshold probabilities.

### 2.2 Patient cohort and hematologic data collection

Because the transcriptome-based analyses primarily reflected tissue-level and microenvironment-related signals, we additionally evaluated pre-treatment peripheral blood parameters as an exploratory approach to assess whether OS might also be accompanied by systemic hematologic immune-related alterations. This analysis was not intended to directly infer tumor microenvironment composition, but rather to provide a complementary systemic perspective.

Patients diagnosed with OS at the First Affiliated Hospital of Guangxi Medical University between 2012 and 2022 were retrospectively included, along with patients diagnosed with cervical spondylosis in the outpatient or inpatient departments during the same period. After data cleaning and eligibility screening for complete pre-treatment CBC variables, 604 OS patients and 600 cervical spondylosis controls were retained for the final hematologic comparison. Clinical data were retrieved from the hospital electronic medical record system, and all patient identifiers were anonymized to protect privacy.

Inclusion criteria comprised patients aged 5 years or older with a confirmed diagnosis of OS or cervical spondylosis and available CBC data prior to initial hospital visit or admission. Patients with acute or chronic inflammatory conditions, recent infections, systemic autoimmune diseases, or those who had received immunosuppressive therapy or chemotherapy within a short period before testing were excluded.

CBC measurements were performed using the hospital’s standardized automated hematology analyzers. The parameters collected included white blood cell count (WBC), red blood cell count (RBC), hematocrit (HCT), mean corpuscular volume (MCV), mean corpuscular hemoglobin (MCH), mean corpuscular hemoglobin concentration (MCHC), platelet count (PLT), platelet distribution width (PDW), neutrophil count and percentage (NEUT and NEUT%), lymphocyte count and percentage (LYM and LYM%), monocyte count and percentage (MONO and MONO%), eosinophil count and percentage (EO and EO%), basophil percentage (BASO%), platelet volume coefficient of variation (CV), and plateletcrit (PCT). Only measurements obtained before any clinical intervention or treatment were included to avoid potential confounding effects on hematologic parameters.

Statistical analyses were performed using SPSS or R software. Continuous variables were assessed for normality. Normally distributed data were compared using Student’s t-test, while non-normally distributed data were compared using the Mann–Whitney U test. Age and sex distributions were additionally summarized to aid interpretation of between-group comparability. Because the cervical spondylosis cohort served as a hospital-based non-malignant comparison group rather than a strictly age-matched healthy control group, the hematologic findings were interpreted with caution, particularly for indices potentially influenced by demographic differences. A two-sided P value < 0.05 was considered statistically significant.

### 2.3 Tissue samples and immunohistochemistry

All tissue samples used in this study were obtained from OS patients at the First Affiliated Hospital of Guangxi Medical University, including 4 paired osteosarcoma tissues and adjacent non-tumor tissues. All specimens were pathologically confirmed and processed immediately after surgery using standardized protocols, followed by formalin fixation and paraffin embedding to preserve tissue morphology and antigenicity.

Immunohistochemistry (IHC) was performed according to a standard protocol to detect APOE expression. Tissue sections were deparaffinized, rehydrated, and subjected to antigen retrieval and endogenous peroxidase blocking. Sections were then incubated with a primary antibody specific for APOE, followed by incubation with a secondary antibody and visualization using diaminobenzidine. All slides were evaluated independently by two experienced pathologists in a blinded manner. APOE expression was semiquantitatively assessed as the percentage of APOE-positive cells among the total number of counted cells in representative microscopic fields and expressed as a positive-cell rate (%). For each specimen, the mean positive-cell rate across the evaluated fields was used for statistical comparison. Because the samples were paired and the sample size was small, comparisons between osteosarcoma and adjacent non-tumor tissues were performed using the Wilcoxon signed-rank test.

### 2.4 Single-cell RNA-seq analysis

To provide cell-type-level support for the tumor microenvironment findings inferred from bulk transcriptomic data, publicly available single-cell RNA-seq data from six primary osteosarcoma samples were analyzed. The analyzed sample accession IDs were TME.GSM9030790, TME.GSM9030791, TME.GSM9030792, TME.GSM9030793, TME.GSM9030794, and TME.GSM9030795. After standard quality-control filtering to remove low-quality cells, a total of 22,997 cells were retained for downstream analysis. Dimensionality reduction and clustering were performed using a standard Seurat workflow. Based on JackStraw analysis, the first 20 principal components were selected for downstream clustering and t-SNE visualization. Cell types were annotated according to canonical marker genes. APOE expression across major cell populations was visualized using feature and dot plots. In addition, CellChat analysis was performed on the annotated single-cell dataset to infer intercellular communication among major cell populations. This analysis was intended as an orthogonal public-data validation to provide cell-type context for the osteosarcoma microenvironment and APOE localization, rather than as a definitive one-to-one validation of all cell-fraction changes inferred from bulk deconvolution.

## 3 Results

### 3.1 Integrated transcriptomic analysis and candidate gene screening

**Figure 1:**
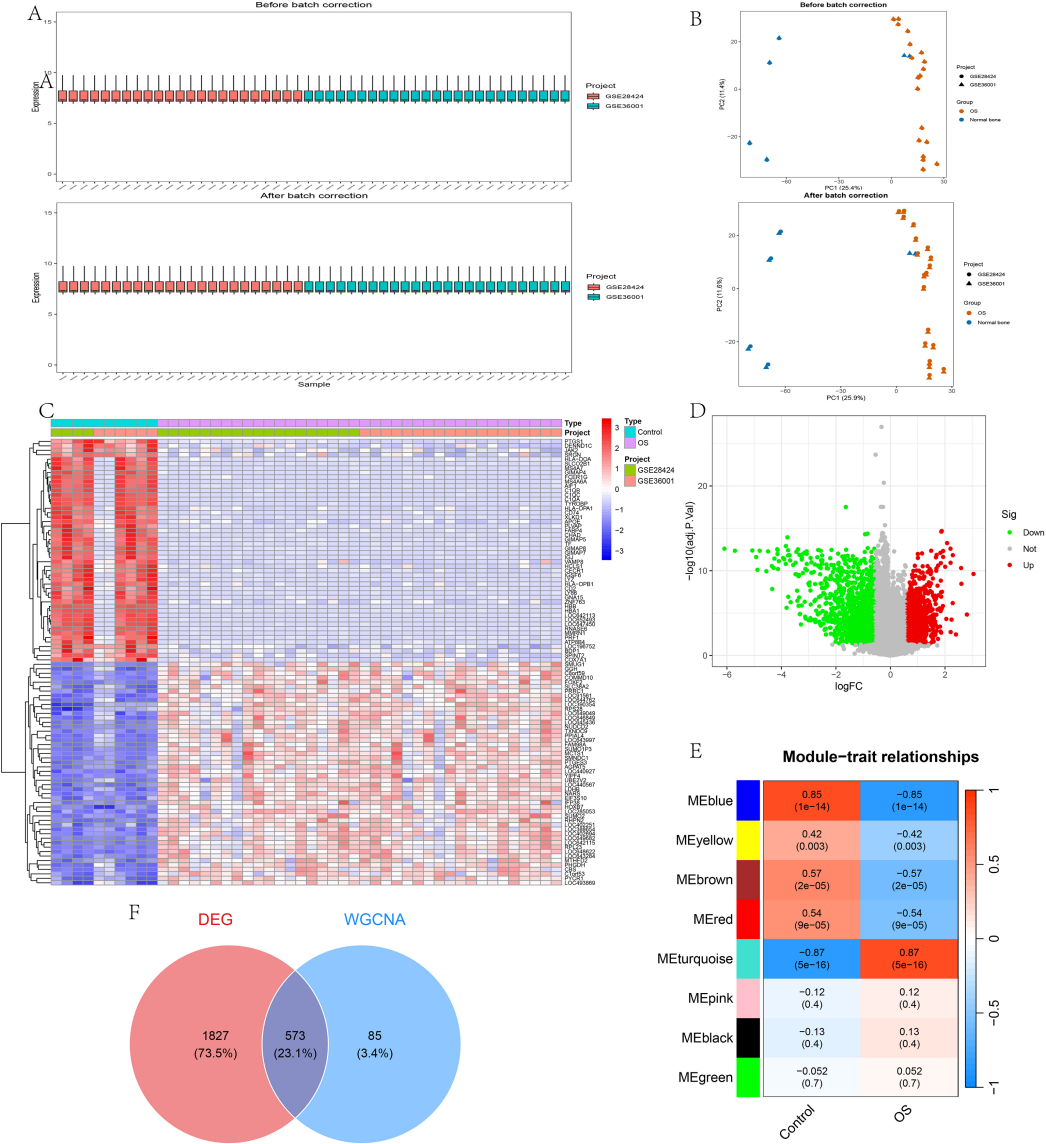
Data integration, batch-effect assessment, differential expression analysis, and WGCNA-based candidate gene screening in osteosarcoma. **(A)** Boxplots of gene expression distributions before and after batch correction in GSE28424 and GSE36001, showing broadly comparable global expression distributions across datasets. **(B)** Principal component analysis before and after batch correction. Biological group (OS vs normal bone) and dataset source (GSE28424 vs GSE36001) are simultaneously annotated to facilitate interpretation of sample distribution and cross-dataset comparability. **(C)** Heatmap of differentially expressed genes (DEGs) between osteosarcoma and control samples, with annotations for sample type and dataset source. **(D)** Volcano plot of DEGs. **(E)** Module–trait relationships identified by weighted gene co-expression network analysis (WGCNA). **(F)** Venn diagram showing the overlap between DEGs and WGCNA-derived candidate genes.

To identify key genes involved in OS, we first analyzed the GEO datasets GSE28424 and GSE36001. After preprocessing the expression matrices, cross-dataset comparability was assessed by boxplots and PCA before and after ComBat adjustment (**Figures 1A, B**). In the revised PCA plots, biological group and dataset source were simultaneously annotated to distinguish sample-type information from cross-dataset structure. The two datasets showed broadly comparable global expression patterns, and no strong dataset-driven separation was observed in the first two principal components either before or after correction, suggesting that large-scale global batch effects were not dominant in these data. Therefore, ComBat was applied as a conservative step to minimize potential residual platform-related variation before downstream integrated analyses. Differential expression analysis was subsequently performed to evaluate biological differences between OS and control samples, and the overall expression patterns of the DEGs were visualized using a heatmap and volcano plot (**Figures 1C, D**). We next conducted WGCNA to identify co-expression modules associated with the OS phenotype, and the module–trait relationships are shown in **Figure 1E**. Finally, genes from the OS-related WGCNA modules were intersected with the DEGs to obtain candidate genes for downstream analyses (**Figure 1F**). These intersected genes were subsequently used for functional enrichment and network analyses.

**Figure 2:**
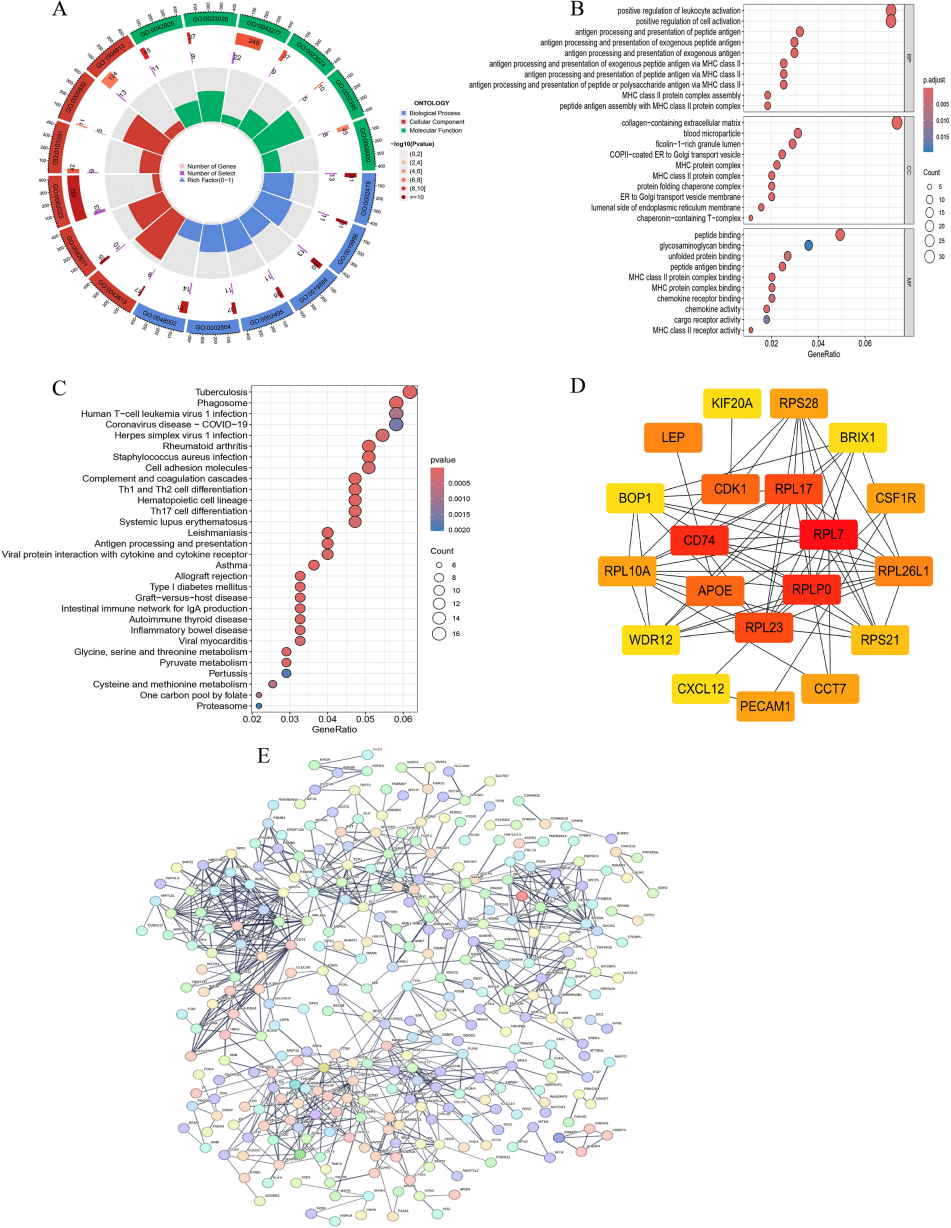
Functional enrichment and protein–protein interaction analyses of candidate genes in osteosarcoma. **(A, B)** Gene Ontology (GO) enrichment analyses of candidate genes. **(C)** Kyoto Encyclopedia of Genes and Genomes (KEGG) pathway enrichment analysis. **(D)** Top hub genes identified from the protein–protein interaction (PPI) network. **(E)** Overall PPI network of candidate genes.

**Figures 2A–C** summarizes the GO and KEGG enrichment analyses of the candidate genes. Among the enriched terms, we noted that several of the most biologically relevant signals were closely related to leukocyte activation, cell activation, and inflammation-associated processes. In particular, GO analysis highlighted positive regulation of leukocyte activation and positive regulation of cell activation, suggesting that immune regulation may represent a major functional axis within the candidate-gene set. In the cellular component category, enrichment in the collagen-containing extracellular matrix further implied a potential link between stromal remodeling and immune-related alterations in the osteosarcoma microenvironment. In the molecular function category, enrichment in peptide binding suggested possible involvement in ligand interaction and signaling regulation. KEGG analysis identified pathways such as Phagosome, Tuberculosis, Human T-cell leukemia virus 1 infection, and Coronavirus disease (COVID-19). Rather than indicating disease-specific relevance, these pathways were interpreted as reflecting shared immune and inflammatory response modules. Because the overall aim of this study was to investigate immune dysregulation in osteosarcoma at both tissue and systemic levels, and because multiple downstream analyses in our workflow specifically addressed immune-cell composition, peripheral hematologic immune indices, and the immune relevance of APOE, we therefore focused subsequent interpretation primarily on the immune-related enrichment signals rather than on all enriched pathways equally. These findings provided the rationale for the downstream immune-infiltration analysis and the subsequent evaluation of neutrophil-related alterations in both tumor tissue and peripheral blood.

To further explore the functional relationships among the candidate genes, a PPI network was constructed using the STRING database and visualized in Cytoscape (**Figure 2E**). Topological analysis identified the top 20 highly connected nodes within the network (**Figure 2D**), including CDK1, RPL17, CD74, RPL7, APOE, RPLP0, and RPL23. These findings provided network-level support for the biological relevance of the candidate genes, although they were not used as the sole basis for final hub-gene prioritization.

### 3.2 Identification of key genes using machine learning algorithms

**Figure 3:**
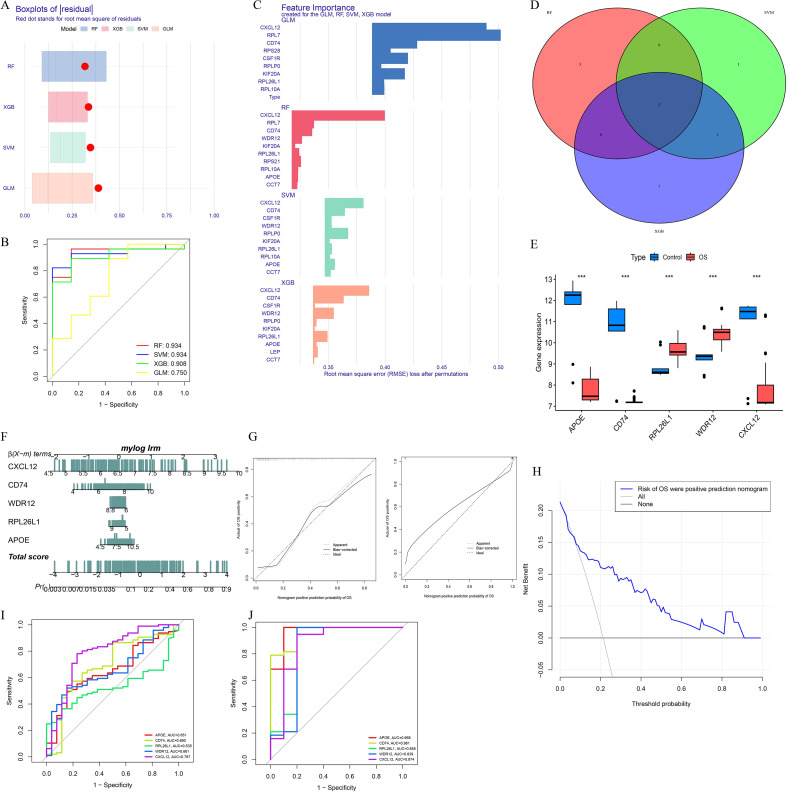
Machine-learning-based identification of hub genes and construction of a gene-based diagnostic model for distinguishing osteosarcoma from control samples. **(A, B)** Performance comparison of RF, XGB, SVM, and GLM models. **(C–E)** Intersection of top-ranked genes and their expression patterns. **(F–H)** Diagnostic nomogram, calibration curves, and decision curve analysis. **(I–J)** ROC analyses of individual hub genes in the training and validation cohorts. “***” indicates P < 0.001.

After normalization and integration of gene expression data from the GSE14359, GSE19276, and GSE21257 datasets, we applied four machine learning algorithms—RF, XGB, SVM and GLM—to identify potential hub genes. **Figure 3A** shows the residual boxplots for each model, illustrating model fitting performance. The diagnostic performance, as measured by area under the curve (AUC), is presented in **Figure 3B**, with RF and SVM achieving the highest AUCs of 0.934, followed by XGB (0.908) and GLM (0.750).

**Figure 3C** displays the top 10 ranked genes from each model. Given the superior diagnostic performance of RF, SVM, and XGB, we intersected the top 10 genes from these three models, resulting in five candidate hub genes (**Figure 3D**): APOE, CD74, RPL26L1, WDR12, and CXCL12. As shown in **Figure 3E**, APOE, CD74, and CXCL12 were significantly downregulated in OS tissues, whereas RPL26L1 and WDR12 were significantly upregulated.

Based on the five candidate hub genes, we constructed a gene-based diagnostic nomogram to distinguish OS from control samples (**Figure 3F**). The model was developed using the integrated training cohort consisting of GSE14359, GSE19276, and GSE21257, whereas GSE28424 and GSE36001 served as independent validation cohorts. Calibration curves for the training and validation cohorts are shown in **Figure 3G**, demonstrating good agreement between predicted and observed classification probabilities in both datasets. Decision curve analysis (DCA) further suggested that the nomogram may provide potential value for transcriptome-based discrimination between OS and control samples (**Figure 3H**).

The diagnostic performance of individual genes in the training cohort was also evaluated, with AUC values of APOE: 0.651, CD74: 0.690, RPL26L1: 0.535, WDR12: 0.661, and CXCL12: 0.787 (**Figure 3I**). In the validation cohort, the AUC values increased substantially, reaching APOE: 0.968, CD74: 0.961, RPL26L1: 0.855, WDR12: 0.839, and CXCL12: 0.874 (**Figure 3J**), indicating that these genes exhibit strong diagnostic potential in an independent cohort and further confirming the robustness and reliability of the nomogram model.

Among the five candidate hub genes, APOE was selected for further validation not solely on the basis of diagnostic performance, but through a combined consideration of its consistent downregulation across datasets, its reported involvement in immune regulation and tumor–immune interactions, and its close alignment with the central theme of this study, namely immune dysregulation in osteosarcoma. Although other candidates, particularly CXCL12 and CD74, may also be biologically important, APOE was considered the most suitable gene for initial tissue-level validation in the current study. The discrepancy between training and validation AUCs for several individual genes may reflect cross-dataset heterogeneity, including differences in sample composition, platform effects, and preprocessing, and therefore these single-gene performance estimates should be interpreted cautiously.

### 3.3 Immune infiltration

**Figure 4:**
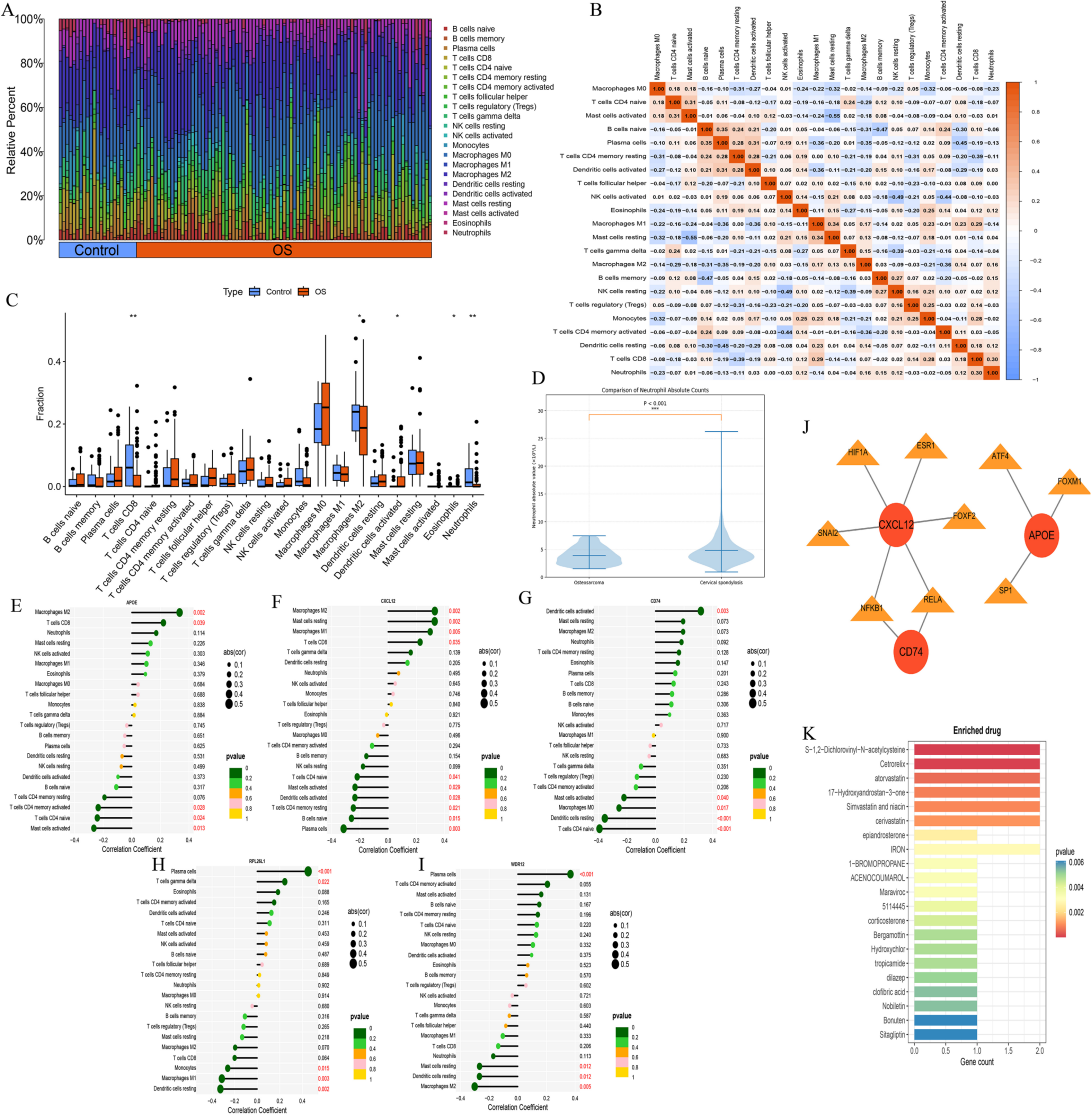
Immune landscape and systemic immune alterations in osteosarcoma. **(A–C)** Immune infiltration profiles. **(D)** Comparison of peripheral neutrophil absolute counts between OS patients and controls. **(E–I)** Correlations between hub genes and immune cells. **(J, K)** Transcription factor network and drug enrichment analysis. “*” indicates P < 0.05 and “**” indicates P < 0.01.

**Figure 4A** presents a bar plot illustrating the abundance of immune cells in each sample. **Figure 4B** depicts the correlations among different immune cell types. Differential expression analysis of immune cells in OS tissues is shown in **Figure 4C**, revealing significant alterations in CD8^+^ T cells, M2 macrophages, activated dendritic cells, eosinophils, and neutrophils, with neutrophils and CD8^+^ T cells notably downregulated in tumor samples.

**Figures 4E–I** illustrate the correlations between the five hub genes identified via machine learning and immune cell populations. APOE was positively correlated with M2 macrophages and CD8^+^ T cells, and negatively correlated with activated memory CD4^+^ T cells, naive CD4^+^ T cells, and activated mast cells. CXCL12 exhibited positive correlations with M2 macrophages, resting mast cells, M1 macrophages, and CD8^+^ T cells, and negative correlations with naive CD4^+^ T cells, activated mast cells, activated dendritic cells, resting memory CD4^+^ T cells, naive B cells, and plasma cells. CD74 was positively correlated with activated dendritic cells, and negatively correlated with activated mast cells, M0 macrophages, resting dendritic cells, and naive CD4^+^ T cells. RPL26L1 showed positive correlations with plasma cells and gamma delta T cells, and negative correlations with monocytes, M1 macrophages, and resting dendritic cells. WDR12 was positively correlated with plasma cells, and negatively correlated with resting mast cells, resting dendritic cells, and M2 macrophages.

### 3.4 Alterations in peripheral blood immune cell profiles in OS

To explore whether the tissue-level immune-related findings might be accompanied by systemic hematologic alterations, we analyzed pre-treatment CBC data from 604 OS patients and 600 hospital-based non-malignant controls with cervical spondylosis. As shown in **Table 1**, the two groups were comparable in age (24.03 ± 14.18 vs. 23.53 ± 14.47 years, P = 0.544) and sex distribution (male, 62.4% vs. 63.3%, P = 0.788).

**Table 1:** Comparison of peripheral blood parameters between osteosarcoma patients and cervical spondylosis controls.

Parameter	Osteosarcoma (n=604)	Cervical spondylosis (n=600)	P-value
Age, years	24.03 ± 14.18	23.53 ± 14.47	0.544
Male, n (%)	377 (62.4%)	380 (63.3%)	0.788
Female, n (%)	227 (37.6%)	220 (36.7%)	
WBC	5.90 ± 2.08	7.74 ± 3.15	<0.001
RBC	4.00 ± 0.76	4.67 ± 0.74	<0.001
HCT	0.34 ± 0.06	0.40 ± 0.06	<0.001
MCV	85.47 ± 8.04	85.42 ± 8.45	0.904
MCH	28.12 ± 3.08	28.29 ± 3.31	0.346
MCHC	328.56 ± 11.86	330.68 ± 12.62	0.003
PLT	266.42 ± 115.67	279.75 ± 105.19	0.037
PDW	0.15 ± 0.03	0.14 ± 0.03	<0.001
Neutrophil absolute value	3.87 ± 1.56	4.83 ± 2.75	<0.001
NEUT (%)	0.65 ± 0.13	0.60 ± 0.11	<0.001
Lymphocyte absolute value	1.34 ± 0.71	2.09 ± 0.91	<0.001
LYM (%)	0.23 ± 0.10	0.29 ± 0.10	<0.001
Monocyte absolute value	0.53 ± 0.85	0.59 ± 0.30	0.083
MONO (%)	0.08 ± 0.06	0.08 ± 0.03	0.043
Eosinophil absolute value	0.14 ± 0.22	0.19 ± 0.23	<0.001
EO (%)	0.02 ± 0.03	0.03 ± 0.03	0.645
BASO (%)	0.00 ± 0.00	0.01 ± 0.00	0.025
CV	0.16 ± 0.03	0.14 ± 0.02	<0.001
PCT	0.22 ± 0.09	0.24 ± 0.09	<0.001

Compared with controls, OS patients showed a significantly lower neutrophil absolute count (3.87 ± 1.56 vs. 4.83 ± 2.75 ×10^9^/L, P < 0.001). However, the neutrophil percentage was slightly higher in the OS group than in controls (0.65 ± 0.13 vs. 0.60 ± 0.11, P < 0.001). Thus, among peripheral neutrophil-related indices, the reduction was more evident in the absolute count than in the relative proportion. The difference in neutrophil absolute counts was further visualized in **Figure 4D**.

In addition, significant between-group differences were observed in multiple hematologic indices, including WBC, RBC, HCT, MCHC, PLT, PDW, lymphocyte-related indices, eosinophil absolute counts, CV, and PCT (**Table 1**), suggesting broad alterations in peripheral blood-cell composition in OS.

### 3.5 Transcription factor regulatory network

**Figure 4J** illustrates the predicted regulatory network between the identified hub genes and their corresponding transcription factors. This analysis was included as an exploratory overview of potential upstream regulatory relationships that may be relevant to gene dysregulation in OS. Although the network highlights several candidate transcription factors that may merit further investigation, these results are hypothesis-generating and were not used as direct mechanistic evidence in the current study.

### 3.6 Drug enrichment

To explore the potential pharmacologic relevance of the identified hub genes, we performed a drug enrichment analysis (**Figure 4K**). Several compounds were predicted to be associated with the hub genes. However, these results should be regarded as exploratory and hypothesis-generating only, as no functional validation was performed in the present study. Therefore, this analysis is presented as supplementary information that may inform future mechanistic or therapeutic investigations.

### 3.7 Immunohistochemistry analysis

**Figure 5:**
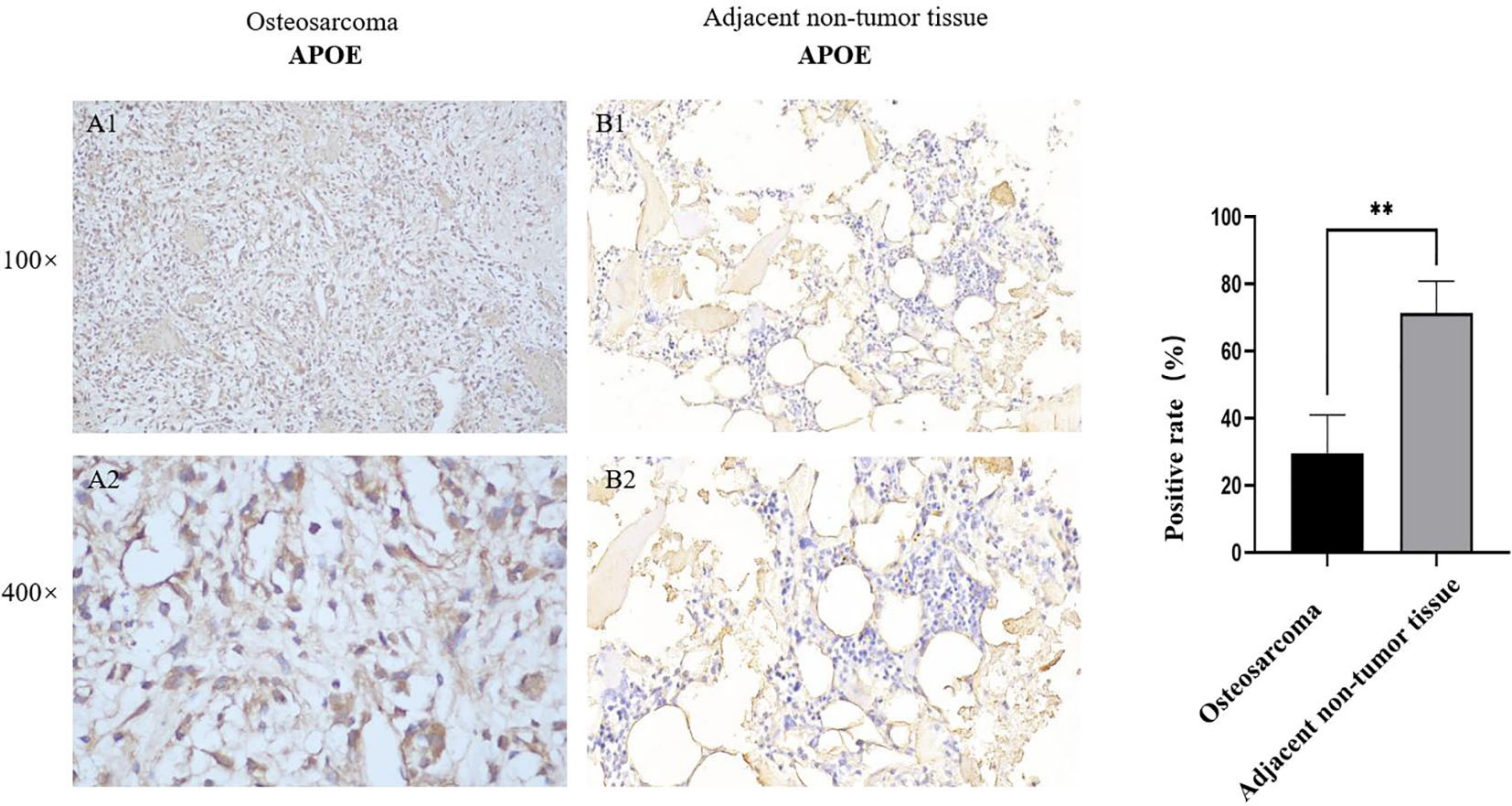
Immunohistochemical validation of APOE expression in paired osteosarcoma and adjacent non-tumor tissues. Representative APOE staining in osteosarcoma tissue **(A1, A2)** and adjacent non-tumor tissue **(B1, B2)** at low and high magnification. The bar plot shows the semiquantitative positive-cell rate (%), defined as the percentage of APOE-positive cells among the total counted cells in the evaluated microscopic fields. APOE expression was lower in osteosarcoma tissues than in the paired adjacent non-tumor tissues. “**” indicates P < 0.01.

**Figure 5** presents the immunohistochemical validation of APOE expression in four paired osteosarcoma and adjacent non-tumor tissue samples. APOE expression was semiquantitatively assessed as the percentage of APOE-positive cells among the total counted cells in representative microscopic fields. The results showed that the positive-cell rate of APOE was significantly lower in osteosarcoma tissues than in the paired adjacent non-tumor tissues. Representative images at low and high magnification are shown in **Figures 5A1–B2**. These findings further support the downregulation of APOE in OS at the tissue level and provide preliminary histopathological evidence for its potential involvement in the osteosarcoma microenvironment.

### 3.8 Single-cell transcriptomic characterization of the osteosarcoma microenvironment

**Figure 6:**
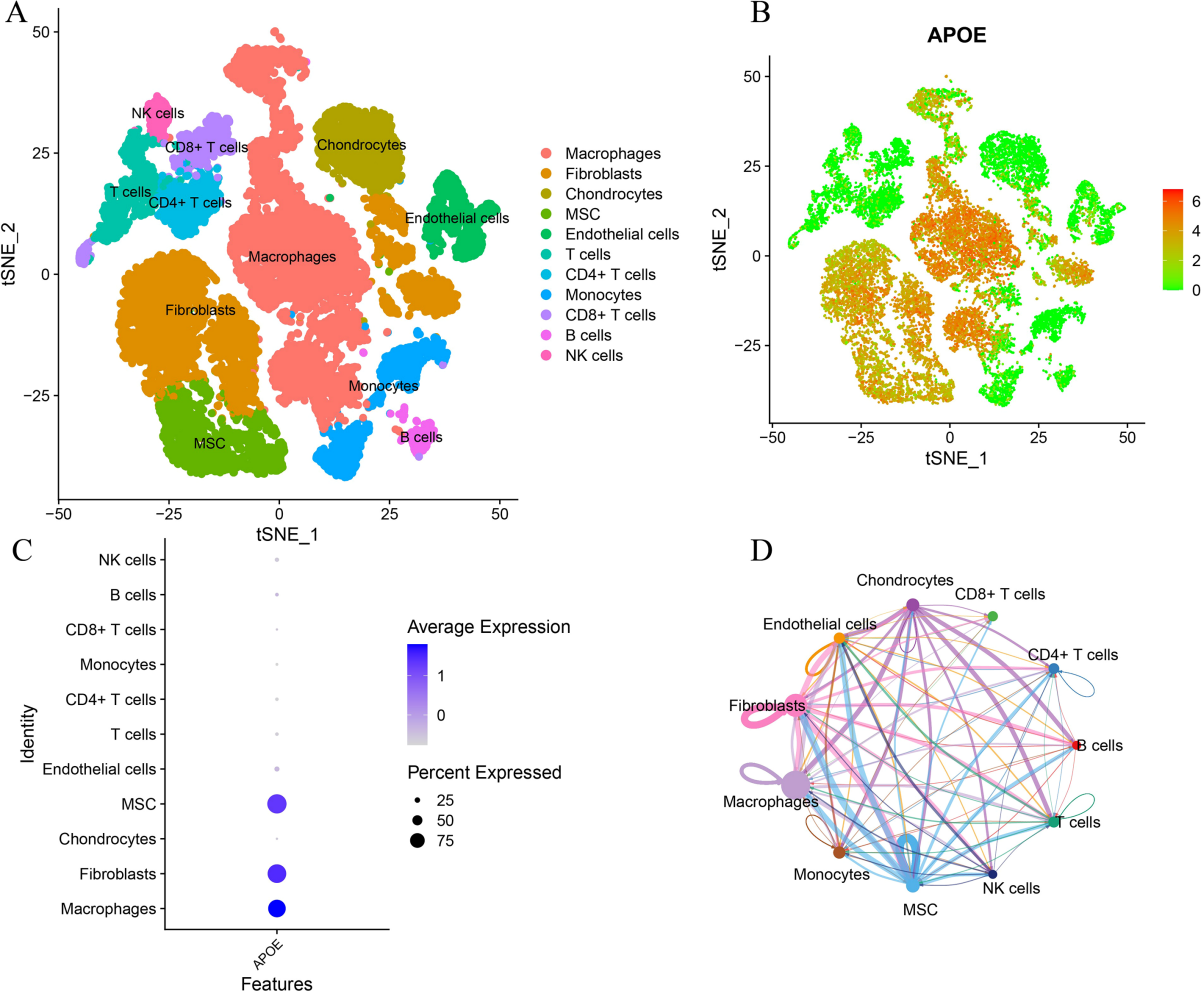
Single-cell transcriptomic characterization of the osteosarcoma microenvironment and cell-type-specific APOE expression. **(A)** annotated t-SNE plot showing 11 major cell populations identified in six primary osteosarcoma samples. **(B)** Feature plot of APOE expression across the single-cell landscape. **(C)** Dot plot showing cell-type-specific APOE expression, with predominant expression in macrophages and lower expression in fibroblasts and MSCs. **(D)** CellChat interaction network illustrating intercellular communication among major cell populations.

To provide cell-type-level support for the bulk transcriptome-based immune analyses, we further analyzed publicly available single-cell RNA-seq data from six primary osteosarcoma samples (TME.GSM9030790–TME.GSM9030795). After quality control, 22,997 cells were retained for downstream analysis. Based on canonical marker genes, 11 major cell populations were identified, including macrophages, fibroblasts, chondrocytes, MSCs, endothelial cells, monocytes, T cells, CD4^+^ T cells, CD8^+^ T cells, B cells, and NK cells (**Figure 6A**). Among these, macrophages and fibroblasts represented prominent cellular compartments within the osteosarcoma microenvironment. APOE expression was not uniformly distributed across cell types; instead, it was predominantly enriched in macrophages, with lower but detectable expression in fibroblasts and MSCs, whereas lymphoid populations showed minimal expression (**Figures 6B, C**). In addition, CellChat analysis suggested extensive intercellular communication among major cell populations, with visually prominent signaling interactions involving macrophages, fibroblasts, endothelial cells, and MSCs (**Figure 6D**). Together, these single-cell findings provide cell-type-level support that APOE is mainly associated with myeloid/stromal compartments in osteosarcoma, thereby complementing the bulk transcriptomic and IHC results.

## 4 Discussion

The primary findings of this study reveal significant downregulation of APOE expression in OS tissues, together with altered peripheral neutrophil-related indices in the clinical cohort. Specifically, OS patients showed lower neutrophil absolute counts, although the neutrophil percentage was modestly higher than that in controls. These findings suggest that peripheral neutrophil alterations in OS may be more complex than a simple uniform decrease and should be interpreted mainly on the basis of the absolute-count difference.

This focus on immune regulation was not based on a single enrichment term alone, but on the convergence of multiple analytical layers in the present study, including immune-related GO/KEGG enrichment, transcriptome-based immune infiltration, peripheral blood immune-cell indices, and single-cell localization of APOE within myeloid/stromal compartments.

IHC analysis further confirmed that APOE expression was significantly reduced in OS tissues, supporting the transcriptome-based findings and strengthening the evidence that APOE is dysregulated in the OS microenvironment (10). Importantly, our additional public single-cell RNA-seq analysis provided orthogonal, cell-type-level support by showing that APOE was predominantly expressed in macrophages, with lower expression in fibroblasts and MSCs, whereas lymphoid populations exhibited minimal expression. This pattern suggests that APOE dysregulation in OS may mainly involve myeloid/stromal compartments rather than being uniformly distributed across all immune-cell populations. Together with the bulk immune-infiltration results, these findings support the view that APOE is closely linked to the immunoregulatory landscape of osteosarcoma.

However, these findings should still be interpreted cautiously. The association between APOE expression and immune-cell infiltration in our study remains correlative, and no direct mechanistic relationship can be established from the current data alone. In particular, the added single-cell analysis mainly provides cell-type context for APOE localization and broad microenvironmental composition, but it does not directly validate every specific population change inferred from bulk deconvolution, such as neutrophil reduction or M2 macrophage enrichment. Therefore, APOE should be regarded as a biologically relevant and high-priority candidate for functional validation rather than as a definitively proven regulator of OS immunity. Future studies should investigate this question using APOE gain- and loss-of-function approaches in OS cell lines, immune-cell co-culture systems, and animal models to determine whether APOE directly affects immune-cell recruitment, cytotoxic lymphocyte activity, or macrophage polarization. In particular, orthotopic or syngeneic osteosarcoma models with APOE modulation, combined with flow cytometry and spatial profiling, may help determine whether APOE plays a causal role in shaping the osteosarcoma immune microenvironment.

Although APOE was selected for further validation, the other identified hub genes may also be biologically relevant in OS. This prioritization should be interpreted as a biologically guided choice for initial validation rather than as definitive evidence that APOE is superior to the other hub genes in all analytical dimensions. CD74 is closely involved in antigen presentation and immune-cell activation, suggesting that its downregulation may indicate attenuated antigen-presenting capacity and reduced immune surveillance in the osteosarcoma microenvironment (11). CXCL12 is a well-known chemokine involved in leukocyte trafficking and stromal–tumor interactions, and its decreased expression may be related to altered immune-cell recruitment in OS (12). In contrast, the functional relevance of RPL26L1 and WDR12 in OS immunity remains less well characterized, but their consistent identification by the machine-learning models suggests that they may also represent potentially important disease-associated genes deserving further investigation (13).

Importantly, computationally inferred neutrophil infiltration in tumor tissue and peripheral blood neutrophil-related indices represent different biological dimensions and therefore should be regarded as complementary rather than interchangeable evidence (7, 14, 15). In the present study, transcriptome-based immune infiltration suggested reduced neutrophil infiltration in OS tissues, whereas the clinical hematologic comparison showed altered peripheral neutrophil-related indices, characterized by lower absolute neutrophil counts but higher neutrophil percentages in the OS group relative to a hospital-based non-malignant comparison cohort. Therefore, the peripheral blood findings should not be interpreted as direct evidence of tumor microenvironment composition.

The discrepancy between our hematologic observations and some prior reports may reflect differences in study design, comparator selection, clinical heterogeneity, disease burden, and the specific neutrophil-related metrics examined. Notably, several previous studies have focused primarily on the prognostic value of neutrophil-related markers within osteosarcoma cohorts, rather than on direct comparison with non-malignant control populations (7, 16, 17). Accordingly, our results should be interpreted as exploratory evidence of systemic hematologic immune-related alterations in OS, rather than as a universal statement that circulating neutrophils are reduced in all osteosarcoma patients.

Furthermore, OS may contribute to altered peripheral neutrophil homeostasis through mechanisms such as bone marrow suppression or alterations in hematopoiesis (18). Chemotherapy, commonly used in OS treatment, is known to cause bone marrow suppression, leading to a decrease in peripheral blood neutrophils (19). This reduction is closely linked to the tumor’s immune evasion and its response to treatment. Chronic systemic inflammation induced by OS may also play a role in neutrophil depletion, potentially through immune exhaustion. Prolonged systemic inflammation has been shown to result in neutrophil depletion, which, in turn, can promote the survival and proliferation of tumor cells (20).

Altered neutrophil-related hematologic indices, particularly lower neutrophil absolute counts, may be associated with the clinical progression, metastasis, and overall survival of OS patients (16). In conclusion, lower peripheral neutrophil absolute counts may serve as a marker of immune dysregulation in OS and may also have potential prognostic relevance. These observations may offer a rationale for future investigation into neutrophil-related pathways in OS, but their therapeutic relevance remains to be established experimentally. Recent studies have suggested that manipulating neutrophil infiltration in the tumor microenvironment could enhance the effectiveness of immunotherapy, offering a promising direction for the treatment of OS (14, 17).

OS exhibits a markedly immunosuppressive tumor microenvironment, which was also reflected in our immune infiltration analysis. Compared with adjacent non-tumor tissues, OS samples showed a significant reduction in CD8^+^ T cells, activated dendritic cells, and neutrophils, accompanied by an enrichment of M2 macrophages (21). This pattern indicates a shift of the tumor microenvironment toward an immunosuppressive state, potentially facilitating immune evasion and tumor progression. The depletion of cytotoxic CD8^+^ T cells and neutrophils may impair anti-tumor immunity (15), whereas the predominance of M2 macrophages could promote tumor growth through the secretion of immunosuppressive cytokines such as IL-10 and TGF-β, consistent with previous reports (22).

This study has several limitations. First, the immune-infiltration analysis based on bulk transcriptomic datasets is inherently observational and does not permit causal inference. In particular, because the comparator group consisted of hospital-based cervical spondylosis patients rather than healthy individuals, the peripheral blood findings should be interpreted as relative differences within this specific clinical comparison framework rather than as definitive normative conclusions for osteosarcoma. In addition, computational deconvolution of immune cells may be affected by tumor heterogeneity, platform differences, and sequencing depth. Although we incorporated publicly available single-cell RNA-seq data to provide cell-type-level context for the osteosarcoma microenvironment and APOE localization, the inferred changes in specific immune-cell populations from bulk deconvolution—particularly neutrophils and M2 macrophages—were not directly validated by dedicated experimental approaches such as multiplex immunofluorescence or flow cytometry in our own cohort. Importantly, computationally inferred immune-cell infiltration in tumor tissue and peripheral blood-cell composition represent different biological dimensions and therefore should not be interpreted as directly equivalent measures. Second, although the clinical cohort was relatively large, the retrospective design may still introduce residual confounding, including unrecognized inflammatory conditions, variability in blood-test timing, and selection bias related to data completeness. In addition, the control group consisted of hospital-based cervical spondylosis patients rather than healthy individuals, which may still influence the interpretation of hematologic differences despite the broadly comparable age and sex distributions between groups. Third, the number of tissue samples used for histological validation was limited to four paired samples and may not fully represent different disease stages or clinical subtypes. In addition, the IHC validation was based on a small number of paired samples and a semiquantitative positive-cell rate rather than an intensity-weighted standardized scoring system such as H-score or IRS; therefore, the tissue-level findings should be interpreted as preliminary histopathological support. In addition, we were unable to robustly assess the relationship between neutrophil-related indices and disease extent, such as metastatic status or tumor burden at diagnosis, because these variables were not uniformly available in the retrospective dataset. Therefore, the present findings should be interpreted as correlative and hypothesis-generating. Larger, multicenter, prospectively designed studies with more appropriate control populations, expanded tissue validation, and independent functional experiments are needed to confirm whether APOE dysregulation and neutrophil-related alterations play causal roles in osteosarcoma immune regulation.

## 5 Conclusion

In this study, we observed altered peripheral neutrophil-related indices, including lower absolute neutrophil counts, in OS patients relative to the hospital-based comparison cohort. Additional public single-cell RNA-seq analysis further localized APOE expression mainly to macrophages, with lower expression in fibroblasts and MSCs, providing cell-type-level support for its involvement in the osteosarcoma microenvironment. Collectively, these findings support the presence of systemic and tissue-level immune dysregulation in OS and suggest potential directions for future mechanistic and translational research.

## Data Availability

The raw data supporting the conclusions of this article will be made available by the authors, without undue reservation.
